# Toxicity Effect of Silver Nanoparticles in Brine Shrimp *Artemia*

**DOI:** 10.1155/2014/256919

**Published:** 2014-01-02

**Authors:** Chinnasamy Arulvasu, Samou Michael Jennifer, Durai Prabhu, Devakumar Chandhirasekar

**Affiliations:** Department of Zoology, Unit of Aquaculture and Animal Tissue Culture, University of Madras, Guindy Campus, Chennai 600 025, India

## Abstract

The present study revealed the toxic effect of silver nanoparticles (AgNPs) in *Artemia nauplii* and evaluated the mortality rate, hatching percentage, and genotoxic effect in *Artemia nauplii*/cysts. The AgNPs were commercially purchased and characterized using field emission scanning electron microscope with energy dispersive X-ray spectroscopy. Nanoparticles were spherical in nature and with size range of 30–40 nm. *Artemia* cysts were collected from salt pan, processed, and hatched in sea water. *Artemia nauplii* (II instar) were treated using silver nanoparticles of various nanomolar concentrations and LC_50_ value (10 nM) and mortality rate (24 and 48 hours) was evaluated. Hatching percentage of decapsulated cysts treated with AgNPs was examined. Aggregation of AgNPs in the gut region of *nauplii* was studied using phase contrast microscope and apoptotic cells in *nauplii* stained with acridine orange were observed using fluorescence microscope. DNA damage of single cell of *nauplii* was determined by comet assay. This study showed that as the concentration of AgNPs increased, the mortality rate, aggregation in gut region, apoptotic cells, and DNA damage increased in *nauplii*, whereas the percentage of hatching in *Artemia* cysts decreased. Thus this study revealed that the nanomolar concentrations of AgNPs have toxic effect on both *Artemia nauplii* and cysts.

## 1. Introduction

Nanotechnology is the development and manufacture of materials in the nanometer size range (at least one dimension less than 100 nm) and their application [1]. Nanoparticles (NPs) have become a part of our daily life, in the form of cosmetics [2], drug delivery systems [3], therapeutics [4], and biosensors [5]. However, little is known about their biodistribution and bioactivity. The various interactions of NPs with fluids, cells, and tissues need to be considered, starting at the portal of entry and then via a range of possible pathways towards target organs. A discipline of nanotoxicology would make an important contribution to the development of a sustainable and safe nanotechnology. Silver nanoparticles have gained much popularity on account of their antimicrobial properties [6, 7]. They are extensively used in detergents and wound dressings, which end up in the environment during waste disposal [8]. The release of silver nanoparticle as discrete particles or as composite colloids, and of Ag+ from various types of textiles [9, 10] and paints used for outdoor facade applications, was observed [11]. However, quantitative data on the release of AgNPs into the aquatic environment and measured environmental concentrations of AgNPs are not currently available. Consequently, the entering of AgNPs into the aquatic environment can only be predicted by models that consider the AgNPs life-cycle from their production till their disposal [12–16]. The most relevant processes that govern the stability and mobility of AgNPs in the aquatic environment are AgNPs agglomeration, aggregation, dispersion, sedimentation, and dissolution. These processes are dependent on the particle physicochemical properties that are in turn influenced by environmental parameters such as pH, temperature, ionic strength, and presence of ligands or natural organic matter [17–19].

*Artemia* (brine shrimp) is zooplankton that is used to feed larval fishes [20]. *Artemia* present one common characteristic, that is, their strong adaptability to hypersaline environments, such as permanent salt lakes, coastal lagoons, and man-made salt pans. They play an important role in the energy flow of the food chain in marine environment [21–24]. *Artemia *use in toxicology poses a reasonable number of answerable questions, namely, practical considerations of laboratory culture and attainment of cyst, ecological relevance, systematic use, and practical conditions of maintenance and sustainability of laboratory conditions of animal model, thus making achievable a sustainable development of *Artemia*-based bioassays [25].

## 2. Materials and Methods

### 2.1. Chemicals

Silver nanoparticles were purchased as a synthetic source from ALDRICH SIGMA CHEMICALS of molecular weight 107.87 g/mL and 0.02 mg/mL concentration diluted in aqueous buffer and containing sodium citrate as stabilizer.

### 2.2. Field Emission Scanning Electron Microscope (FESEM) with Energy Dispersive X-Ray Spectroscopy (EDX)

The morphology and size of silver nanoparticles were measured by field emission scanning electron microscope (HITACHI SU6600). A minute drop of nanoparticles solution was cast on aluminum foil and subsequently dried in air before transferring it to the microscope. An energy dispersive X-ray detection instrument (EDX) (HORIBA 8121-H) was used to examine the elemental composition of the sample.

### 2.3. *Artemia* Cyst Collection, Processing, and Hatching Procedure

*Artemia* cysts were collected from salt pan of Kelambakkam, Chennai, using net of pore size 150–200 micrometer. Cysts were cleaned and they were filtered and spread on the paper which absorbs water and kept for shadow drying for one night [20]. Decapsulation is the removal of the outer membrane of a cyst called the chorion by dissolution in sodium hypochlorite, without affecting the viability of the embryo. Before hatching procedure the cysts were decapsulated using sodium hypochlorite. Approximately 2 g of the precleaned cysts was incubated in 1 L seawater in a conical plastic contained with graduations at 30 ± 1°C. 1,500 lux light intensity was provided continuously by a fluorescent lamp. Aeration was maintained by a small line extending to the bottom of the hatching device from an aquarium air pump. Under these conditions, *Artemia *cysts were hatched within 24 hours.

### 2.4. Mortality Rate of *Artemia nauplii*

The acute toxicity was determined by measuring the adverse effect of various concentrations of silver nanoparticle on brine shrimp *Artemia nauplii* growth, survival and mortality under intermittent flow-through conditions. The study commenced with <24-hour-old *nauplii* and continued and exposed for 24 and 48 hours. Experiment was performed in 12-well plate. In each well containing 2 mL of 33 ppt of saline water along with control (without silver nanoparticles) about 10 *nauplii* were transferred with the addition of desired concentration of AgNPs such as 2 nM, 4 nM, 6 nM, 8 nM, 10 nM, and 12 nM, respectively [26]. Each test concentration along with the control should be carried out for three replicates in 12-well plate. The experimental setup was allowed to remain 24 hours in darkness and *nauplii* were counted after incubation time. The LC_50_ value after 24 hours and percentages of mortality after 24 and 48 hours for various test concentrations of silver nanoparticles were determined and compared with the control. Results were tabulated and plotted as a graph.

Percentage mortality was calculated by following the formulae

(1) %Mortality=Number  of  dead  Artemia  naupliiInitial  number  of  live  Artemia  nauplii×100.

### 2.5. Hatching Percentage of *Artemia* Cyst

Brine shrimp *Artemia *cysts were collected and processed and were used for testing the percentage of hatching. Before performing the test, the cysts were decapsulated using sodium hypochlorite for 15 minutes. Experiment was performed in 12-well plates. And in each well containing 2 mL of 33 ppt in saline water along with control (without AgNPs), about 20 *Artemia* cysts were transferred with the addition of desired concentration of AgNPs such as 2 nM, 4 nM, 6 nM, 8 nM, 10 nM, and 12 nM. Each test concentration along with the control was carried out in three replicates in 12-well plate. The experimental setup was allowed to remain 24 hours in shaker for aeration and the experimental set up was exposed to light to provide favorable environment for the cyst to hatch out. Hatched cysts were counted after the incubation period of 24 hours. Percentages of hatching for various test concentrations of silver nanoparticles were determined.

Percentages of hatching for various test concentration of silver nanoparticle were calculated by the following formula:

(2) $$\begin{array}{c} H\%=\frac{N}{C+N}\times 100, \end{array}$$

where *H* is the hatching percentage, *N* is the number of hatched cysts, including the umbrella stage, and *C* is the decapsulated full cysts.

Results were tabulated and plotted as a graph.

### 2.6. Morphological Variations of *Artemia nauplii*

The morphological variations in *Artemia nauplii* treated with various concentrations of silver nanoparticles and control alone were observed using inverted phase contrast microscope (RADICAL).

### 2.7. Apoptosis Analysis: Acridine Orange Staining

To investigate the role of apoptosis by AgNPs toxicity, acridine orange staining of nanoparticle-treated *Artemia nauplii* was performed. *Artemia nauplii* treated with various concentrations of silver nanoparticles were gently placed in the cavity slides. 10 *μ*L of acridine orange stain from the stock of 5 *μ*g/mL was added to the *nauplii* for 20 minutes at room temperature. *Artemia nauplii* were washed in phosphate buffer saline (1X PBS, pH 7.4). Stained samples were observed under inverted fluorescence microscope (RADICAL).

### 2.8. Single-Cell Gel Electrophoresis (Comet Assay)

A comet assay was performed to determine the DNA damage [27]. Approximately 200 *Artemia nauplii* were collected 24 hours after treatment from the control and LC_50_ value and were pooled for a comet assay. Treated organisms were placed in 1 mL of phosphate-buffered saline (PBS) containg 20 mM ethylene diamineni tetraacetic acid (EDTA) and 10% dimethyl sulfoxide (DMSO) and disintegrated mechanically by mincing. Cell suspension was precipitated by vortexing and then immediately mixed with 100 *μ*L of 1% low melting agarose (LMA) at 39°C and spread on a fully frosted microscopic slide precoated with 200 *μ*L of 1% normal melting agarose. After the solidification of agarose, slides were covered with another 75 *μ*L of 0.5% LMA and then immersed in lysis solution (2.5 M NaCl, 10 mM Na-EDTA, 10 mM Tris, 1% Trion X100 and 10% DMSO, pH 10) for 1 hour at 4°C. The slides were then placed in a gel electrophoresis apparatus (containing 300 mM NaOH and 10 mM Na-EDTA, pH 13) for 40 minutes to allow unwinding of DNA and the alkali labile damage. Next, an electrical field (3000 mA, 25 V) was applied for 20 min at 4°C to draw the negatively charged DNA toward an anode. After electrophoresis, slides were washed thrice for 5 min at 4°C in a neutralizing buffer (0.4 M Tris, pH 7.5), followed by staining with 75 *μ*L of propidium iodide (40 *μ*g/mL) and then the slides were observed using fluorescence microscopy.

## 3. Results

### 3.1. FESEM/EDAX Investigation

**Figure 1:**
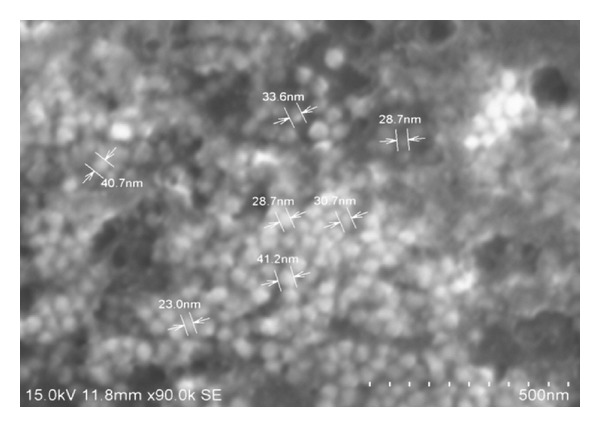
Photomicrograph showing spherical shape of silver nanoparticles using field scanning electron microscope.

**Figure 2:**
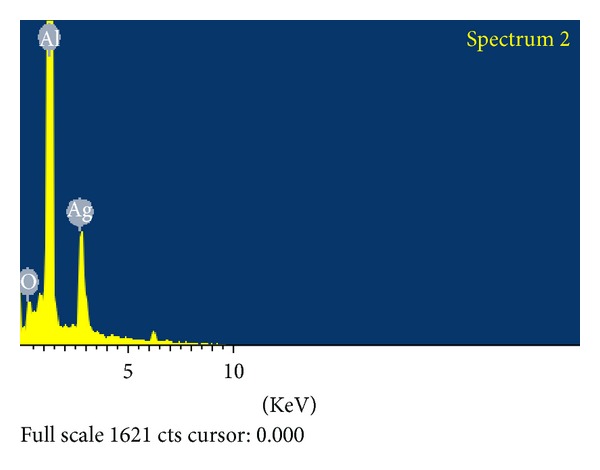
Energy dispersive X-ray spectrum of silver nanoparticles.

Field emission scanning electron microscope was used to investigate the morphology and size of the silver nanoparticles. The AgNPs obtained with the sodium citrate reduction method were predominantly spherical with diameters ranging from a few nanometers to 30–40 nm (Figure 1). EDAX spectrum recorded on the experimental sample is shown as three peaks located between 2 KeV and 10 KeV. Those maxima are directly related to the silver characterized lines K and L. The maximum peak located on the spectrum at 0.2 KeV clearly coming from aluminum. The second maximum peak located on the spectrum at 0.3 KeV clearly indicates comes from silver. Third peaks located at 0.1 KeV are connected with the oxygen characteristics line (Figure 2).

### 3.2. Mortality Rate of *Artemia nauplii*

The nanoparticle aggregates to elevated levels such that the guts were filled with particles showing significant mortality within 24 hours of exposure. The results were found to be in such a way that in the control the mortality was about 6% which was negligible. In minimum concentration of 2 nM the mortality was 16% and as the concentration increases from 4 nM, 6 nM, and 8 nM the mortality was about 33%, 36.6%, and 43%. About 53.3% and 66.6% of the population of *Artemia nauplii* were found to be dead as the concentration increased to the maximum in the test concentrations of 10 nM and 12 nM. The LC_50_ value was obtained around 10 nM concentration.

**Figure 3:**
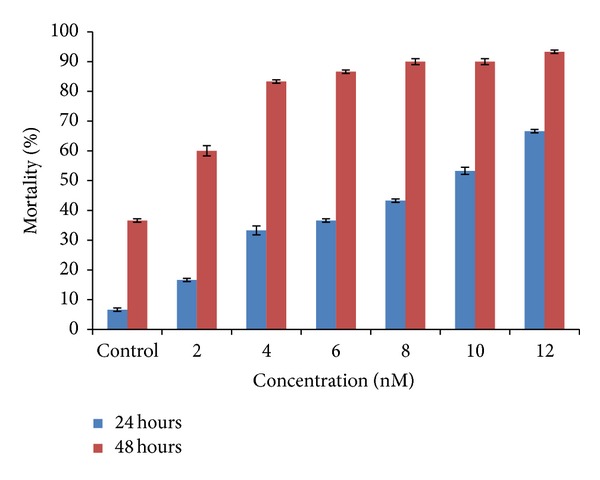
Bar diagram shows results for mortality rate (24 and 48 hours) of brine shrimp *Artemia nauplii* treated with various nanomolar concentrations of silver nanoparticles.

And further extended exposure to 48 hours did induce high mortality. After 48 hours the mortality rate was twice the results of the 24-hour mortality rate and even in 2 nM concentration about 60% mortality was observed and in concentration between 4 nM, 6 nM, and 8 nM the mortality increased to 83.3%, 86.6% and 90% which was twice the mortality rate of 24-hour exposure. And the mortality was found to be above 90% at 10 nM and 93% at 12 nM concentration. However, these effects were most likely due to the lack of food uptake since the guts were completely filled with the aggregates of silver nanoparticles (Table 1 and Figure 3).

### 3.3. Hatching Percentage of *Artemia nauplii*

**Figure 4:**
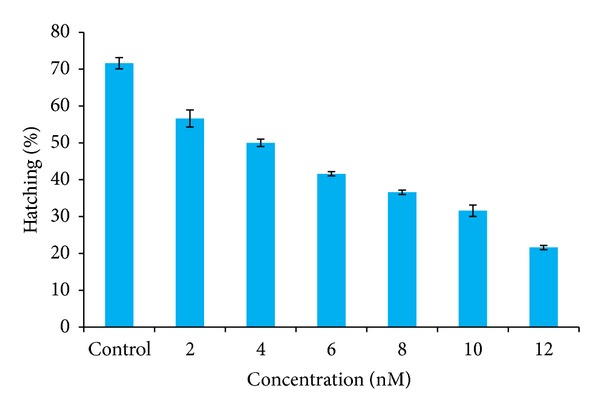
Hatching percentage of *Artemia* cysts treated with various nanomolar concentrations of silver nanoparticles.

This study reveals that the silver nanoparticles have an effect not only on the alive animal but also on cysts. It is due to the diffusion of silver nanoparticles through the smooth outer layer of the decapsulated cysts. The results were found after 24 hours to be in such a way that in the control the percentage of hatching was more than 70% which was normal. In minimum concentration of 2 nM the percentage of hatching was 56% and as the concentration increases from 4 nM, 6 nM, and 8 nM, the percentage of hatching was about 50%, 41.6%, and 36%, correspondingly. The percentage of hatching was very low as 31.6% and 21.6% as the concentration increased to the maximum such as 10 nM and 12 nM. Hence the results revealed that as the concentration of the silver nanoparticles increases, the effect of toxicity increases and the hatching percentage of *Artemia* cysts decreases (Table 2 and Figure 4).

### 3.4. Morphological Variation of *Artemia nauplii* Treated with Silver Nanoparticles

The aggregations of silver nanoparticles inside the gut of *Artemia nauplii* were clearly observed under the phase contrast microscope and the images were photographed. As *Artemia* generally exhibits nonselective filter feeding behavior, it consumes all particles that are below 50 microns in size. The amount of aggregation not only depends on the amount of concentration, but also depends on the amount of consumption of nanoparticles by each individual animal in various concentrations.

**Figure 5:**
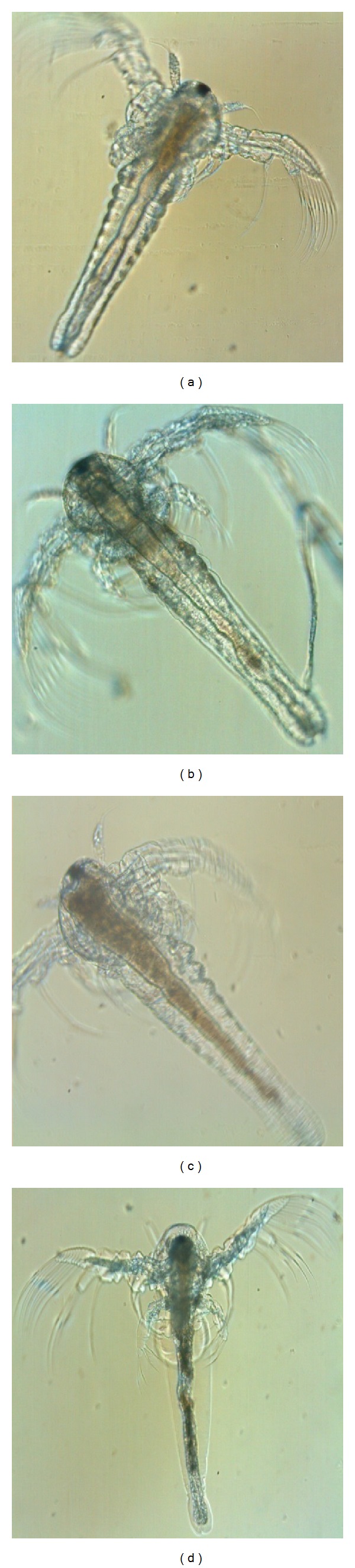
Morphological variations of *Artemia nauplii* treated with silver nanoparticle observed using inverted phase contrast microscope (20x magnification): control (a), 2 nM concentration of AgNPs (b), LC_50_ concentration (10 nM) of AgNPs (c), and 12 nM concentration of AgNPs (d).

In this study the *Artemia nauplii* were treated with various nanomolar concentrations of AgNPs such as 2 nM, 4 nM, 6 nM, 8 nM, 10 nM, and 12 nM and after 48 hours of treatment they were observed under the phase contrast microscope and results were photographed. The results showed that in control the animal did not show any trace of aggregation; the mouth parts and the gut region appeared clear (Figure 5(a)). In minimum concentration (2 nM) the aggregation of silver nanoparticles was found around the mouth parts and some regions of the gut (Figure 5(b)), whereas in higher concentration (10 nM), the entire gut region was accumulated with silver nanoparticles (Figure 5(c)). And in maximum concentration (12 nM) the gut was completely filled with silver nanoparticle; due to the effect of high toxicity of silver nanoparticles the tissue of the animal started to degrade (Figure 5(d)).

### 3.5. Apoptosis Analysis: Acridine Orange Staining

**Figure 6:**
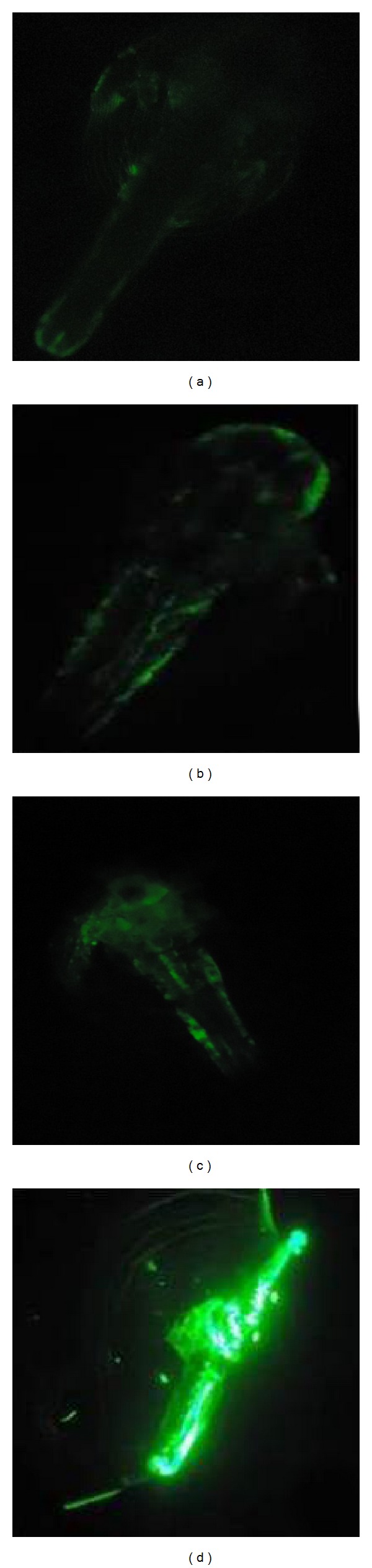
Acridine orange stained *Artemia nauplii* treated with silver nanoparticles observed using fluorescence microscope (20x magnification): control (a), 2 nM concentration of AgNPs (b), LC_50_ concentration (10 nM) of AgNPs (c), and 12 nM concentration of AgNPs (d).

The observations were in accordance with *in vitro *toxicity study on silver nanoparticle, where higher concentrations had more apoptotic cells and necrotic cells than lower concentrations of silver nanoparticle. In order to study the occurrence of apoptosis, *Artemia nauplii* treated with various concentrations of silver nanoparticles were stained using acridine orange and observed under the fluorescence microscope. At higher concentration such as 12 nM, *Artemia nauplii* exhibited bright green spots all over the body (Figure 6(d)). In lower concentrations only few green spots were visible in the animal body (Figures 6(b) and 6(c)). And in control there was no appearance of green spots in animal body, as there were no apoptotic cells damaged due to silver nanoparticle (Figure 6(a)). This is due to the condition that as the concentration increases the amount of cell damage increases and the emission of fluorescence by acridine orange stain is proportional to the amount of necrotic cells due to the effect of toxicity of silver nanoparticles. Hence in the animal body the region that emits fluorescence reveals the condition of apoptosis due to toxicity of silver nanoparticles.

### 3.6. Single Cell Gel Electrophoresis Analysis

**Figure 7:**
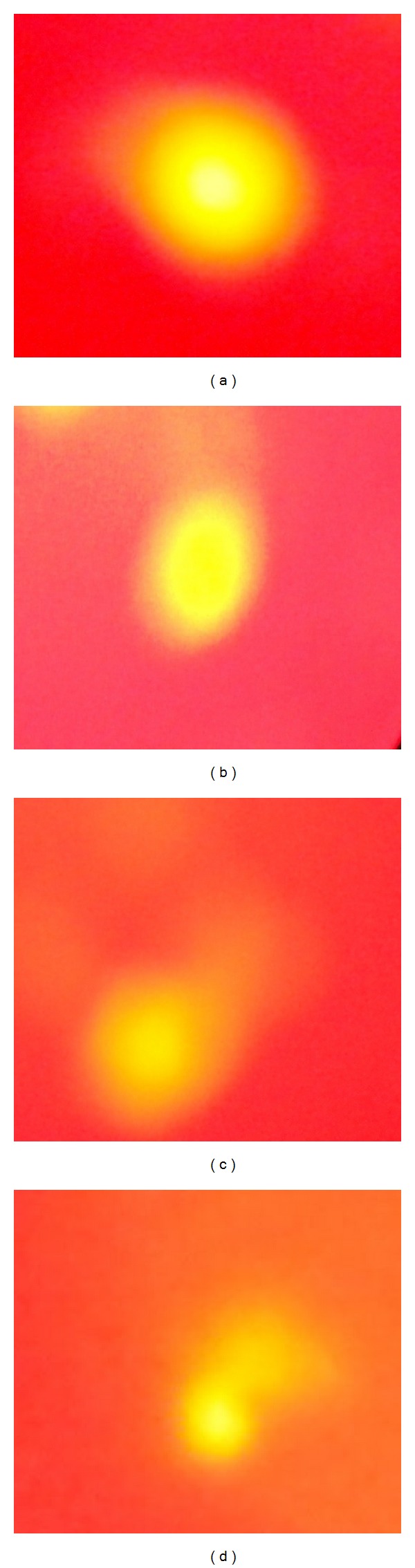
Fluorescence microscope images (comets) of the propidium iodide stained DNA of *Artemia nauplii* cells exposed to LC_50_ concentration of 10 nM AgNPs (c), minimum concentration of 2 nM AgNPs (b), and maximum concentration of 12 nM AgNPs (d) as compared with the control (a) (40x magnification).

In this present study the control result shows that relatively undamaged cells give comets consisting of a compact head with or without a very short tail, indicating double-stranded DNA. Comets originating from damaged cells (LC_50_ and 12 nM concentration) have a distinct head with a tail. The most basic way of viewing the data from the comet assay is the distribution of cells according to the percentage of DNA in tail moment. The tail DNA of the LC_50_ and 12 nM concentration results was compared with appropriate control. It can be seen from Table 3 that control did not evoke a significant effect on tail DNA damage of about 3.2%. In minimum concentration of 2 nM the tail DNA damage was observed to be 9.9% and LC_50_ concentration (10 nM) evoked an increase in percentage of tail DNA damage of 25.5% and at the maximum concentration of 12 nM the increase in tail DNA damage of about 47.9% was compared with the control. Thus in this regard the results of our study clearly indicate that silver nanoparticle used as commercial formulation of above 12 nM concentration will cause high level of DNA damage to the *Artemia *due to the toxicological effect (Table 3 and Figure 7).

## 4. Discussion

Nanoparticles deserve special attention, because compounds in this miniature size range have chemical properties that differ from those of their larger counterparts [28]. *Artemia *is one of the most valuable test organisms available for marine ecotoxicity testing as it is widely used as a nutritious live food source to the larvae of a variety of marine organisms, which makes them the most convenient, least labor-intensive live food available for aquaculture and will be impacted greatly by the release of nanoparticles into aquatic systems because of their high levels of interaction with the environment through nonselective filter feeding [29].

In this present study the bioassay lethality test obtained LC_50_ value at 10 nM concentration among the various concentrations such as 2 nM, 4 nM, 6 nM, 8 nM, 10 nM, and 12 nM [30]. The previous results suggest that the toxicity of silver nanoparticles to aquatic species depends on a concentration-dependent manner [31].

In the present findings, the nanoparticle aggregates to elevated levels of guts that were filled with particles showed significant mortality within 24 hours exposure. The results were found to be in such a way that in the control the mortality was about 6% which was negligible. More than half of the population of *nauplii* was found to be dead as the concentration increased to the maximum concentrations of 10 nM and 12 nM. And after 48 hours the mortality rate was more than 60% in lower concentrations (<6 nM) and 90% of mortality in higher concentrations of 10 nM and 12 nM. However, these effects were most likely due to the lack of food uptake since the guts were completely filled with the aggregates of silver nanoparticles. The previous study has reported a reduction in hatching percentage of *Artemia franciscana *cysts exposed to mercury at a concentration as low as 10 nM Hg. The emergence and hatching curves crossed later in the mercury-exposed cysts than in the controls, with the delay being longer in cysts exposed to higher concentrations. This study reveals that the silver nanoparticles have effect not only on the alive animal but also on cysts [32]. Hence in this present study the results revealed that as the concentration of the silver nanoparticles increases (10 nM and 12 nM) the effect of toxicity increases and the percentage of hatching of the *Artemia* cysts decreases to 20%.

Accumulation of TiO_2_ NPs was performed qualitatively on each group at the conclusion of the exposure under a phase contrast microscope equipped with a digital camera. The ingested TiO_2_ particles appeared as a long strip of particles suggesting that even larger aggregates of TiO_2_ formed inside the guts [33]. And in the present study the aggregation of silver nanoparticles inside the gut of *Artemia nauplii* was clearly observed under the phase contrast microscope. The results showed that in control the animal did not show any trace of aggregation; the mouth parts and the gut region appeared clear. And in maximum concentration (12 nM) the gut was completely filled with silver nanoparticle; due to the effect of high toxicity of silver nanoparticles the tissue of the animal started to degrade.

The toxicity study of silver nanoparticles in Zebra fish embryos revealed that the acridine orange staining to study apoptosis showed no significant staining in control embryos whereas the Ag-BSA and Ag-starch treated embryos showed green fluorescent spots on the body, which could be explained using the decomposition of body parts [31]. In the present findings acridine orange staining to study apoptosis showed no significant staining in control *nauplii* as there is no such effect of toxicity of silver nanoparticles. The region in the animal body that emits fluorescence reveals the condition of apoptosis due to toxicity of silver nanoparticles. This is due to the condition that as the concentration increases the amount of cell damage increases and the emission of fluorescence by acridine orange stain is proportional to the amount of apoptotic cells due to the effect of toxicity of silver nanoparticles. And the maximum emission of fluorescence was observed in 12 nM concentration.

The single-cell gel electrophoresis was done in human lymphocytes treated with pesticides such as isomalathion and compared with the control. Isomalathion evoked an increase in comet length at concentration of 200 *μ*M and the increase was 72% as compared with the control [34]. Sun-Young et al. [35] who have reported comet assay have performed an *in vitro* system from aquatic species; BPA exerts a genotoxic effect on *D. magna* and *C. tentans*, given that DNA strand breaks increased in both species exposed to this compound, whereas NP- and CP-induced DNA damage occurred only in *C. tentans.* In this present study the control result shows that relatively undamaged cells give comets consisting of a compact head with or without a very short tail, indicating double-stranded DNA. Control did not evoke a significant effect on tail DNA damage of about 3.2%. In minimum concentration of 2 nM the tail DNA damage was observed to be 9.9% and LC_50_ concentration (10 nM) evoked an increase in tail DNA damage of 25.5% and at the maximum concentration of 12 nM the increase in tail DNA damage of about 47.9% was compared with the control. Thus, in this regard the results of our study clearly indicate that silver nanoparticle used as commercial formulation of the above 12 nM concentration will cause high level of DNA damage to the *Artemia* due to the toxicological effect.

Understanding the potential impacts of these particles can help in identifying the most appropriate nanotechnology that will preserve the marine aquatic environment while also advancing medical and environmental technology. This result also is attributed to our limited knowledge regarding the overall implications of nanoparticles on marine ecosystems as the nanoparticles have proved to be detrimental in this acute toxicity test; its effect in the long run is to be carefully analyzed. Considering that the smaller nanoparticles turned out to be toxic in the present test, it cannot be excluded that the overall toxicological potential will increase in the future. Hence it can be concluded that the uncontrolled and unobserved release of these nanoparticles, either as byproducts or medical wastes, could have a large negative consequence on the aquatic, terrestrial organisms and also on humans. Hence, studies on the fate and effects of nanoparticles in the environment and in the living organisms are needed to more clearly define the benefits and potential risks of this promising technology.

## Figures and Tables

**Table 1:** The result for mortality rate (24 and 48 hours) of brine shrimp *Artemia nauplii* treated with various nanomolar concentrations of silver nanoparticles.

Parameter	Concentration levels in nanomolar (nm)	Initial number of *Artemia nauplii *	Number of *nauplii* dead after 24 hours	Number of *nauplii* dead after 48 hours	% of mortality after 24 hours (mean ± SD)	% of mortality after 48 hours (mean ± SD)
Mortality rate after 24 and 48 hours	Control	30	2	11	6.60 ± 0.57	36.60 ± 0.57
	2	30	5	18	16.60 ± 0.57	60.00 ± 1.73
	4	30	10	25	33.30 ± 1.52	83.30 ± 0.57
	6	30	11	26	36.60 ± 0.57	86.60 ± 0.57
	8	30	13	27	43.30 ± 0.57	90.00 ± 1.00
	10	30	16	27	53.30 ± 1.15	90.00 ± 1.00
	12	30	20	28	66.60 ± 0.57	93.30 ± 0.57

**Table 2:** Hatching percentage of *Artemia* cysts treated with various nanomolar concentrations of silver nanoparticles.

Parameter	Concentration levels in nanomolar (nm)	Initial number of *Artemia *cyst	Number of cysts hatched after 24 hours	Number of cysts unhatched after 24 hours	Hatching percentage (mean ± SD)
Hatching efficiency after 24 hours	Control	60	43	17	71.66 ± 1.52
	2	60	34	26	56.66 ± 2.30
	4	60	30	30	50.00 ± 1.00
	6	60	25	35	41.60 ± 0.57
	8	60	22	38	36.66 ± 0.57
	10	60	18	42	31.66 ± 1.52
	12	60	13	47	21.66 ± 0.57

**Table 3:** Comet assay parameters using CASP software of comet assay.

Comet assay parameter	Control	Minimum concentration (2 nM)	LC_50_ value (10 nM)	Maximum concentration (12 nM)
Length of head	171	57	39	189
Length of tail	19	14	9	56
Length of comet	190	71	48	245
Head DNA (%)	96.738	90.0932	74.404	52.0968
Tail DNA (%)	3.26201	9.90679	25.596	47.9032
Tail movement	0.619783	1.38695	2.30364	26.8288
Olive tail movement	2.61229	3.0077	4.99172	25.4624
